# Wild Ungulates Constitute the Basis of the Diet of the Iberian Wolf in a Recently Recolonized Area: Wild Boar and Roe Deer as Key Species for Its Conservation

**DOI:** 10.3390/ani13213364

**Published:** 2023-10-30

**Authors:** Isabel Barja, Álvaro Navarro-Castilla, Lorena Ortiz-Jiménez, Ángel España, Roberto Hinojosa, David Sánchez-Sotomayor, Ángel Iglesias, José España, Sergio Rubio-Sánchez, Santiago Martín-Romero, Juan Vielva, Fernando Horcajada-Sánchez

**Affiliations:** 1 Unidad de Zoología, Departamento de Biología, Universidad Autónoma de Madrid, 28049 Madrid, Spain; 2 Research Centre in Biodiversity and Global Change (CIBC-UAM), Universidad Autónoma de Madrid, 28049 Madrid, Spain; 3 SIGNATUR, Carretera de la Sierra, 45, Villavieja del Lozoya, 28739 Madrid, Spain; 4 SIGNATUR, C/Asunción Castell, 22, 28739 Madrid, Spain; 5 Centro de Investigación, Seguimiento y Evaluación del Parque Nacional de la Sierra de Guadarrama, TRAGSA, 28740 Madrid, Spain; 6 Consejería de Medio Ambiente, Vivienda y Agricultura, Centro de Investigación, Seguimiento y Evaluación del Parque Nacional de la Sierra de Guadarrama, 28740 Madrid, Spain

**Keywords:** *Canis lupus signatus*, domestic ungulates, wild ungulates, Iberian wolf, recolonization

## Abstract

**Simple Summary:**

We conducted a study in central Spain to understand the dietary habits of the Iberian wolf. Our aim was to evaluate the extent to which they prey on domestic ungulates. We analyzed the composition of their diet by examining prey hairs found in 671 wolf scat samples collected between 2017 and 2021. The wolves predominantly consumed wild ungulates rather than domestic ones. Among their preferred prey were wild boar and roe deer. Although their diet varied with seasons, years, and forest regions, a preference for wild ungulates over domestic ones remained consistent.

**Abstract:**

The Iberian wolf (*Canis lupus signatus*) is recolonizing historical distribution areas after decades of absence. As in other human-dominated landscapes, finding a balance to protect this species by favoring recolonization and mitigating human–wildlife conflicts is a challenge. Since wolves are often generalist opportunistic predators, we studied their diet composition in central Spain to evaluate the consumption of domestic ungulates and provide reliable data that could help local authorities to deal with the current wolf–cattle ranchers conflict and coexistence. Diet composition (% prey occurrence, % prey ingested biomass) was analyzed through the identification of prey hairs present in 671 scats collected between 2017 and 2021. The wolves fed more on wild ungulates (82% occurrence) than domestic ones (18%). Wild boar (*Sus scrofa*, 44% occurrence) and roe deer (*Capreolus capreolus*, 35%) were the most consumed prey. The wolves positively selected these two species. The wolves’ diets varied between seasons, years, and forest regions, but a diet based on wild ungulates predominated over domestic ones. Food niche breadth showed variations depending on seasons and years. Preserving the availability and diversity of wild ungulates may favor reducing livestock attacks and would be an achievable goal that would help to conserve this species and reduce conservation conflicts.

## 1. Introduction

The Grey wolf (*Canis lupus lupus*) is an example of a carnivore with a completely disappeared population in some regions of Europe in the 18th century [1,2], due to direct persecution and prey abundance decrease [3], which has been recolonizing semi-desert and agricultural lands in many industrialized countries in recent years [4]. This European recolonization has been favored by endowing the wolf with a strict protection status from Council Directive 92/43/EEC of the EU Habitats Directive, the conservation strategies of the European Commission such as the European Life Program (European Commission, 2020), and the habitat restoration strategies of some countries [5,6,7], constituting a relevant milestone in the rewilding process [8].

Wolves in Spain were rarely observed in the 20th century and reached their lowest numbers in the 1970s. Protective measures were initiated, leading to the expansion of the wolf population in the northeastern mountains and the recolonization of the southeast of Spain [9,10,11]. The EU Habitats Directive classified wolf populations south of the Duero River under strict protection in Annex IV, with populations north of the river enjoying a more flexible Annex V status [12,13]. Protection measures for wolves differ on each side of the Duero River due to variations in livestock damage [13].

Despite the Iberian wolf not meeting the criteria for “vulnerable” status, the Spanish Government chose to maintain Annex IV protection across the country. Consequently, wolves were included in the list of protected species, and hunting was banned nationwide in September 2021. Exceptions for hunting may be considered if it is demonstrated that other “preventive or wildlife protection” measures have been “adequately” implemented and proven “ineffective” (Order TED/980/2021, RD 239/2011).

Wolf damage to livestock is a constant source of socioeconomic conflict according to some authors [14,15,16]. However, this problem must be considered to be a conservation challenge, since the wolves are not knowingly antagonists in conflict [17]. Livestock consumption (17.8–38.9% occurrence) was demonstrated by dietary studies carried out at the end of the 20th century in the northwestern mountains (north of the Duero River), which are highly populated and intensively used areas where livestock in extensive grazing conditions habitually and the presence of wolves is considered to be high [18,19,20,21]. Recent studies, also carried out north of the Duero River, have placed the consumption of livestock between 10.6 and 62.3% of occurrence, varying noticeably between areas [22,23]. However, the recolonization of the southern Duero River is currently being studied and yielding mixed results. For example, an investigation carried out in central Portugal determined that the wolf’s diet depended on domestic livestock by more than 90%, a result that the authors related to the low diversity and density of wild ungulates [6]. Conversely, results from other research conducted in central Spain (Segovia) revealed that, at higher elevations, cattle were subjected to increased attacks, irrespective of wild prey abundance [7]. In any case, wolf attacks substantially increased to the south of the Douro River from 2007 to 2017, while the increase was only moderate in the north. In 2017, for example, >73% of all attacks (*n* = 1989) occurred in the south (Junta de Castilla-León 2017).

Overall, the wolf’s diet is broadly influenced by the structure of prey communities. Studies performed in several European countries have shown a diversity of species in their diet: red deer (*Cervus elaphus*), roe deer (*Capreolus capreolus*), and wild boar (*Sus scrofa*) in Poland [24]; roe deer and wild boar in Italy [25]; predominantly moose (*Alces alces*) and Eurasian beaver (*Castor fiber*), European badger (*Meles meles*), and brown hare (*Lepus europaeus*) as a more sporadic consumption in the Scandinavian Peninsula [26]; roe deer, red deer, wild boar, mouflon (*Ovis orientalis*), and brown hares in Germany [27]. In some parts of the world, the grey wolf feeds on smaller prey species such as rodents, birds, and invertebrates [28,29]. Additionally, the species can be an opportunistic predator depending on the group and body size of the prey [30,31]. The wolf feeds mainly on medium and large ungulates that coexist in its distribution range [32], becoming skilled in the selective hunting of a particular species depending on its availability in each habitat [22,33]. Furthermore, diet is also influenced by various factors such as the genetic structures of populations, prey ecology, climate change, recreational hunting, and agricultural policies [34,35,36,37]. It should be noted that the wolf, as a top predator, plays a fundamental role in maintaining balance in its ecosystem, since it modulates the abundance of mesocarnivores and large herbivores. Thus, an uncontrolled increase in ungulates due to removal of the top predator from the ecosystem can lead to a loss of biodiversity [3]. If, on the other hand, the population of wild ungulates was to be drastically reduced, wolves could become interested in more abundant and easily preyed upon domestic prey, leading to a human–wildlife conflict [38,39].

In the Iberian Peninsula, food resource availability is distributed in a variable way, mainly influenced by anthropogenic uses of the land that are carried out in each area [40,41,42]. For this reason, the feeding habits of the wolf are highly variable depending on the area studied [22,23]. Wolves coexist with humans in Spain, as in other western European countries [42,43]. Although wolves tend to avoid anthropic areas, they benefit from resources associated with humans, such as livestock [44]. Predation on domestic livestock is often associated with areas where wild ungulates’ abundance has been relatively low for decades [45]. However, a study conducted on the north face of the Central System Mountain range did not find an association between a greater abundance of wild ungulates and a reduction in attacks on livestock [7]. Nowadays, the wolf is expanding towards the southern face of the Central System Mountain range, located in Comunidad Autónoma de Madrid [46]. These are areas recently recolonized, where more relaxed extensive livestock farming practices are carried out and where protection against predators is scarcer than in other areas where wolves have inhabited for years [6]. Therefore, the objective of this study was to examine the feeding habits of the wolf by analyzing its diet composition, the diversity of prey in its habitat, and niche breadth in Sierra de Guadarrama National Park, Sierra del Rincón, and the surroundings (located to the south of the Duero River) using a non-invasive methodology by collecting fecal samples. The hypotheses and predictions proposed were:

(i) Wolves are expected to select wild ungulates [47,48,49,50].
(ii) Wolves are expected to feed on the most abundant species [2,32]. The wild boar, known for its large litters [51], is expected to be the primary wild prey choice in line with European studies [27,52].
(iii) Environmental factors reported in previous studies [53,54] are anticipated to influence the wolf diet in the study area, with season and year potentially playing roles.
(iv) We anticipate variations in wolves’ feeding habits among the forest regions in the study area [36,55].

## 2. Materials and Methods

### 2.1. Study Area

**Figure 1:**
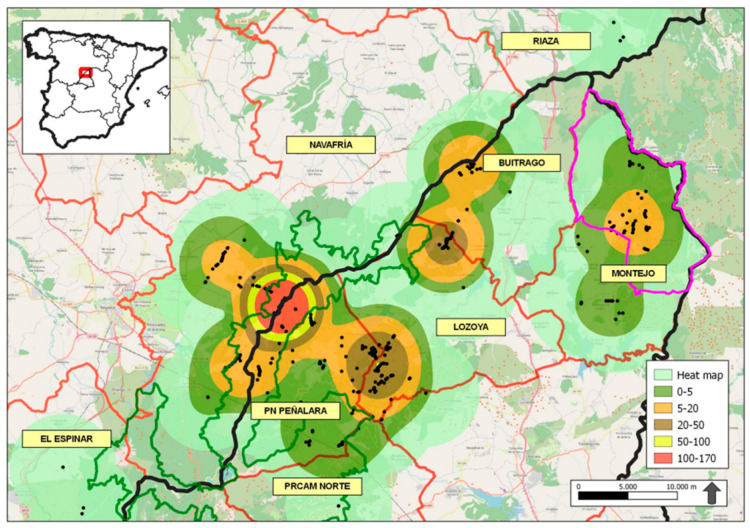
Scats’ densities in forest regions of the study area using Kernel density technique. The colors represent varying densities of collected scats. Red lines mark the forest regions, black lines separate Castilla y León (northwest) from Madrid (southeast), green lines represent Sierra de Guadarrama National Park, and pink lines represent Sierra del Rincón Biosphere Reserve.

We conducted the study in the Sierra de Guadarrama National Park (PNSG, declared by the Spanish Law 7/2013, of 25 June), Sierra del Rincón Biosphere Reserve (Designated by UNESCO on June 29 2005, and expanded in 2022), and the bordering areas (Figure 1). This area covered the territories of several packs of Iberian wolves (Figure 1), a protected species (Law 42/2007) whose conservation is a priority (Directive 97/62/EC). The sampled surface was a mountain range with an extension of 100,775 ha presenting strong slopes and discontinuities, as well as numerous perennials and temporary watercourses. In this area, the average annual temperature ranged from −3.2 °C to 22.4 °C, with annual average rainfall records of 1223 mm (AEMet, www.opendata.aemet.es, accessed on 30 December 2021). So, the climate was described as a continental Mediterranean climate, with dry and temperate summers and cold and humid winters. The study area contained different forest regions, delimited by geographic, ecological, forestry, and socioeconomic criteria and established for the adequate planning and execution of the actions that articulate the management of mountains, forests, and cattle trails (Consejería de Medio Ambiente y Ordenación del Territorio 2007). The forest regions included in the study area were: PN Peñalara, Lozoya, Buitrago, Montejo, PRCAM Norte, El Espinar, amd Navafría y Riaza.

Scotch pine (*Pinus sylvestris* L.) is the predominant forest species (35.4%) between 1200 and 1900 m, unlike the vegetation located at 1100 m, where Pyrenean oak (*Quercus pyrenaica* Willd.; 15.8%) is the most abundant species. In addition to high mountain pastures (23.9%), the study area also has an undergrowth (24.9%) formed by *Cytisus oromediterraneus C. purgans* auct. Non (L.) Spach, Common juniper (*Juniperus communis* L.), Common holly (*Ilex aquifolium* L.), and *Adenocarpus hispanicus* (Lam.) DC. The predominant wild fauna species that are susceptible to be preyed upon by wolves were: wild boar, roe deer, and mountain goat (*Capra pyrenaica*). The ungulates’ species abundance in the area included densities ranging from 3 to 5 ind/km^2^ for wild boars, 3–6 ind/km^2^ for roe deer, and high mountain enclaves with 15–36 ind/km^2^ of mountain goats (ungulate census of the National Park Sierra de Guadarrama 2021–2022, Comunidad de Madrid). However, domestic ungulates were also present in the study area, especially in summer, which takes advantage of pastures coming from a multitude of livestock huts such as cattle, goat (*Capra aegagrus hircus*), sheep (*Ovis orientalis aries*), and horse. The number of domestic ungulates in the area was 100,793 animals, of which 58,454 individuals corresponded to cattle and the rest to goats and sheep [INE 2020].

### 2.2. Collection of Fecal Samples

The collection of fecal samples in the field is a non-invasive and affordable technique that allows for the collection of many samples to perform a qualitative and quantitative diet analysis [22]. The collection of wolf fecal samples was carried out by establishing 15 itineraries along forest trails and firebreaks, places where wolves move and deposit their scats, either with a marking function or as simple excretion [56,57,58]. Since the probability of defecation at cross-roads is higher [56,59], these were also included in the sampling itineraries. In each itinerary, the presence of the species was recorded using signs of its activity (scats, tracks, and/or scratches). Wolf scat was collected by qualified researchers with extensive experience in wolf tracking and wolf scat identification. In general, wolf scat was distinguished from that of medium-sized carnivores and small dogs by its length (>20 cm) and diameter (>2.5 cm). Additionally, to differentiate it from large dogs, scat with the presence of hair and the absence of visible kibble remains was selected. The surveys were carried out on foot and the average length of each itinerary was 3.12 ± 0.44 km (range between 2.47 and 4.60 km). The samplings were carried out monthly for five years (2017–2021), obtaining a total of 671 scats in individual bags, which were identified with a numerical code and recorded using a portable GPS device [60]. The samplings were refrigerated in the laboratory until their subsequent analysis.

### 2.3. Identification of Wolf Prey Species

The diet of the wolves was determined from the analysis of the hair found in the scats collected in the study area. To do this, from m5 to 8 hairs strands were collected from each fecal sample to be washed in a Petri dish with soap and water. After rinsing with water, the hair was left drying on filter paper. The hairs selected from each sample were identified at the species level based on their macroscopic and microscopic characteristics [60]. The macroscopic characteristics, comprising the coloration, shape, length, and thickness of the hair, were observed in a binocular magnifying glass (model Olympus TL2 SZ30, Olymws Opticalco GMBH, Hamburg, Germany). For the correct identification, a comparative study was carried out with the hair of known species from the personal collection of Dr Isabel Barja. The microscopic characteristics (shape, arrangement, margin, and distance between margins of the scales) corresponded to the cuticular pattern of the hair (which varies between species [61]). To observe these cuticular patterns, the dry hair was fixed on a slide spraying a thin and homogeneous layer of hairspray. Each hair was placed, leaving a free end that would facilitate the detachment of the hairspray layer after 10–15 min, enough time to leave the cuticular pattern imprinted on a microscope slide. The cuticular patterns were observed using an optical microscope (Olympus CX41 model) attached to a camera (Color view); we took photographs at different magnifications (10×, 20×, and 40×) with the Soft Imaging system five software (AnalySIS getIT 5.0). The prey species identification was conducted with a mammalian hair atlas [62] and a manual on the macroscopic and cuticular patterns of mammalian hairs from the Iberian Peninsula published by Barja et al. [61].

### 2.4. Mapping Using Kernel Densities

A Kernel density map was made to differentiate the areas with the highest concentrations of wolf scats within the study area, considering the number of collected scats. This way, we could know the possible number of reproductive packs of Iberian wolves in the study area, which is useful for the management of a newly recolonized area. Zub et al. [63] showed the overlaps of the home ranges of 4 wolf packs in Poland, comparing areas comprising 75% of fecal mark locations (plotted using the Kernel method) with the distribution of radiolocations. The Kernel density was calculated by quantifying the relationships of points within a radius of influence by analyzing the patterns of a specific data set. The place of the occurrences was recorded by the means of a coordinate system that allowed for a count of all the points within a region of influence to be weighted by the distance of each one from the place of interest. The density of each region of the study area was calculated via interpolation. Interpolation made it possible to build a continuous surface of the variables (a smoothed surface), inferring the spatial variation of the variable for the entire study area, even in regions where the process had not generated any real occurrence, allowing for the verification of possible data trends [64,65,66].

### 2.5. Statistical Analysis

The results are shown as frequency and percentage of occurrence, and ingested biomass. The frequency of occurrence of the prey species was determined by counting the number of scats that presented hair of each prey species. The ingested biomass (in kg and %) was calculated based on the average weight of each prey species (Table 1) and using the equation of Floyd et al. [67], revised and adjusted by Weaver [68] (see Appendix A), already used in studies on wolf diets in the Iberian Peninsula [6,69,70].

Subsequently, we ran a goodness-of-fit chi-square test (χ^2^) to verify the adjustment between the observed and expected frequencies of the consumed prey species hypothesis. In addition, we used contingency tables to evaluate differences in the relative frequencies of the prey species in relation to the seasonality and years. We used the Pearson’s χ^2^ test (in 2 × 2 tables, where df. = 1, we applied Yates’ continuity correction) for cases in which less than 20% of the expected frequencies in the table had less than 5 records and for cases in which more than 20% of the expected frequencies in the table had less than 5 records; additionally, we used the Monte Carlo exact test (Fisher’s exact statistic was used in 2 × 2 tables and in the rest of the cases the χ^2^ statistic).

Additionally, we calculated the Shannon diversity index to estimate dietary diversity according to seasonality and years (see Appendix A). We tested significant differences between pairs of Shannon indices using Hutcheson’s *t*-statistic. Hutcheson’s *t*-test is a modified version of the classical *t*-test that provides a way of comparing two samples using the variance of the Shannon index [71].

We estimated the niche breadth of the wolf in terms of diet resources according to the frequency of occurrence of prey consumed and the biomass ingested over the seasons and years. We used the Levin’s food niche breadth Index (FNB) [72] (see Appendix A) to quantitatively measure specialization in the composition of the wolf’s diet.

Finally, we calculated the Ivlev’s electivity index modified by Jacobs [73] to assess whether the wolves selected prey positively or negatively (see Appendix A). This index was applied to evaluate the selection of prey throughout the study area and, secondly, to evaluate the selection among domestic ungulates in terms of forest regions. The level of significance to reject the null hypothesis was *p* < 0.05. Statistical tests were carried out using SPSS v.23.00 (SPSS Inc., Chicago, IL, USA).

**Table 1:** Composition of Iberian wolf diet for 5 years (2017–2021) in Sierra de Guadarrama National Park and surroundings based on 671 scats. The ingested biomass (kg) was calculated using body masses obtained from the literature [22,69,74,75,76].

		Prey Occurrence		Ingested Biomass		Prey Mean Mass (Kg)		
Prey		N	%	Kg	%	Adult	Youth	Mean
Wild ungulates	Roe deer	233	34.7	500.7	13.8	24.5	7.0	15.8
	Red deer	2	0.3	10.1	0.3	90.0	25.0	57.5
	Wild boar	294	43.9	1006.3	27.7	75.0	22.0	48.5
	Mountain goat	21	3.1	69.7	1.9	61.0	11.0	36.0
Total		550	82.0	1586.7	43.7			
Domestic ungulates	Cattle	55	8.2	1927.2	53.0	750.0	115.0	432.5
	Domestic goat	28	4.2	47.5	1.3	26.3	5.0	15.7
	Sheep	15	2.2	26.8	0.7	28.5	5.0	16.8
	Horse	2	0.3	49.7	1.3	550.0	60.0	305.0
	Unidentified livestock	21	3.1	-	-	-	-	-
Total		121	18.0	2051.0	56.3			
Total		671	100.0	3637.8	100.0			

### 2.6. Ethic Information

The research methodology adhered strictly to non-invasive techniques, and this study was conducted in full accordance with the laws and regulations established by the Spanish Government.

## 3. Results

### 3.1. General Remarks

The analysis of 671 scats showed that the wolves consumed more wild ungulates compared to domestic ones (Table 1). On the one hand, the differences in the consumption of different prey species were statistically significant in relation to the percentage of occurrence (χ^2^ = 1282.56; df = 8; *p* = 0.001; *n* = 671). Specifically, the wild ungulates most consumed were wild boar and roe deer (χ^2^ = 476.69; df = 3; *p* = 0.001; *n* = 550; Table 1). Among domestic ungulates, cattle were the prey most predated (χ^2^ = 64.08; df = 4; *p* = 0.001; *n* = 121; Table 1). On few occasions, the small amount of hair and its fragmentation in the sample led to doubts between two domestic species. Consequently, to calculate the biomass ingested (kg and %), we did not consider the occurrence obtained for these unidentified domestic samples. Overall, the biomass percentage provided by domestic ungulates was slightly higher (Table 1).

Ivlev’s index showed that the wolves positively selected the wild boar (D = 0.92) and roe deer (D = 0.89) and avoided the mountain goat (D = −0.27) and domestic ungulates (cattle: D = −0.84; sheep and goat: D = −0.80). There was a higher consumption of roe deer from 2017 to 2019 compared to wild boar, but from 2020 to 2021, wild boar was more frequently found in the wolves’ scats (roe deer frequency in scats: mean = 56.2; SD = 47.42), (wild boar frequency in scats: mean = 43.2; SD = 33.95). Furthermore, wild boar represented a greater biomass contribution to wolves’ diets, with 186,6 kg on average; SD = 146.62, while roe deer contributed 95.73 Kg on average; SD = 80.80 on an annual basis.

### 3.2. Seasonal Trends

**Figure 2:**
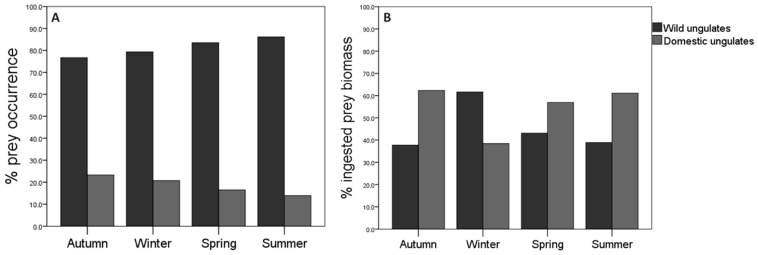
Consumption of wild and domestic ungulates in relation to seasons: (**A**) % prey occurrence; (**B**) % ingested biomass. Sample size in autumn: *n* = 189; winter: *n* = 111; spring: 200; and summer: *n* = 137.

The consumption of wild ungulates was greater than that of domestic ungulates in all seasons. On the one hand, the highest percentage of occurrence for wild ungulates was in summer, while the lowest percentage was in autumn, contrary to the occurrence trend of domestic ungulates (Figure 2A). The percentage of occurrence for roe deer was the highest with respect to the rest of the species in all seasons except in autumn, when wild boar was the predominant species. Regarding domestic ungulates, cattle was the most frequent species in all seasons (see Appendix B). Seasonal differences in the percentages of occurrence for the different prey species were statistically significant (χ^2^ = 66.07; df = 24; *p* = 0.001; *n* = 637; see Appendix B). On the other hand, the ingested biomass of wild ungulates was higher in winter than in the rest of the seasons, while the ingested biomass corresponding to domestic ungulates was higher in autumn compared to other seasons (Figure 2B). The wild boar contributed the most biomass to the wolf’s diet in winter, while cattle did this in the rest of the seasons (Table 2). When interpreting these results, it is important to consider that the percentage of occurrence may be more accurate than the percentage of ingested biomass, as the weight of the entire prey does not necessarily indicate that wolves consume it entirely.

The diversity in the diet of the wolf varied according to season, being higher in autumn (H′ = 1.42), followed by spring (H′ = 1.27) and summer (H′ = 1.22), and lower in winter (H′ = 1.10). The seasonal differences were statistically significant both between autumn and winter (t = 2.85; df = 196; *p* = 0.01) and between autumn and summer (t = 1.98; df = 294; *p* = 0.05). Between autumn and spring, no significant differences were observed (t = 1.66; df = 380; *p* = 0.09).

The Food Niche Breadth (FNB), calculated according to the frequency of occurrence for prey species in the collected scats, had a relatively small variation according to seasons (see Appendix B). Considering two types of ungulate prey (wild vs. domestic), the wolves’ niche adjusted to a diet specialized in wild ungulates in all seasons. Yet, predation on domestic ungulates increased, with a marginal widening in autumn and winter (B standardized = 0.31 and 0.21), while the wolves’ diet was more focused on wild ungulates in spring and summer (B standardized = 0.26 and 0.24). However, when considering four wild prey species (roe deer, wild boar, red deer—Cervus elaphus—and mountain goat), we observed a narrower FNB in winter. On the other hand, when considering four domestic species (cattle, sheep, domestic goat, and horse), the wolf showed the narrowest FNB in summer compared to the other seasons, in which it turned to a more generalist strategy. FNB calculated according to the amount of biomass (kg) also varied according to seasons. Considering two types of ungulate prey (wild/domestic), FNB showed a generalist diet in all seasons (B < 0.6). However, the values of FNB obtained showed a specialist diet when they were calculated considering the four wild species (B < 0.6). The same occurred when considering the four domestic species.

### 3.3. Annual Trends

**Figure 3:**
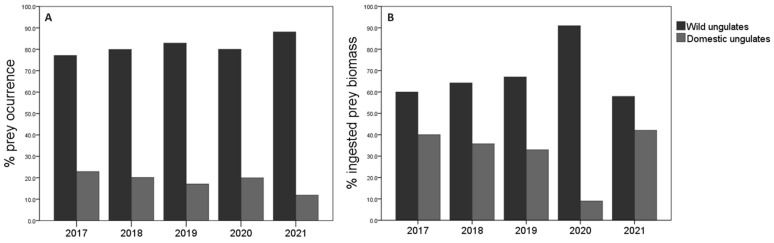
Consumption of wild and domestic ungulates in relation to years: (**A**) % prey occurrence; (**B**) % prey biomass ingested. Sample size 2017: *n* = 109; 2018: *n* = 169; 2019: *n* = 287; 2020: *n* = 30; and 2021: *n* = 42.

The consumption of wild ungulates by wolves was higher compared to domestic ungulates in all years, considering that the abundance of each species in the study area did not change substantially during the evaluated period. On the one hand, the consumption of wild ungulates increased over time until 2019, yet decreased for the two following years. In contrast, domestic ungulate consumption decreased from 2019 to 2021, being higher in 2017, which corresponded with the first year of the study (Figure 3A). According to the percentage of occurrence, roe deer predominated in 2017, 2018, and 2019, while wild boar did in 2020 and 2021. Regarding domestic ungulates, the consumption of goat and cattle was similar in 2017; later, cattle was the most preyed upon domestic species during the 2018–2020 period, while in 2021, the goat was more preyed upon (see Appendix B). The percentage of occurrence for the different prey species according to the years was statistically significant for roe deer (χ^2^ = 327.61; df = 32; *p* < 0.001; *n* = 637; see Appendix B). On the other hand, regarding the ingested biomass, the values provided by wild ungulates were higher in all years. Overall, the biomass from wild ungulates was similar between years, but a maximum was detected in 2021. Conversely, although the biomass provided by domestic ungulates was also similar between years, a noticeable decrease occurred in 2020 (Figure 3B). The wild prey species that contributed most to the wolf diet in terms of biomass were wild boar and roe deer, while cattle was the most consumed domestic ungulate according to the percentage of biomass in all years except 2020 (see Appendix B).

The year also influenced the diversity in the wolf´s diet. The greatest diversity of diet was shown in 2019 (H′ = 1.39), unlike 2021, when the lowest diversity was noted (H′ = 1.02). Overall, the diversity was similar in the rest of the years (2017: H′ = 1.17; 2018: H′ = 1.14; 2020: H′ = 1.15). Significant differences were observed in the diversity of the wolf diet between 2017 and 2019 (t Hutcheson = 2.37; df = 186; *p* = 0.02), 2018 and 2019 (t Hutcheson = 2.90; df = 319; *p* = 0.01), 2019 and 2020 (t Hutcheson = 2.07; df = 46; *p* = 0.04), and 2019 and 2021 (t Hutcheson = 3.02; df = 61; *p* = 0.01).

The Food Niche Breadth (FNB) of the wolf was calculated according to the frequency of occurrence for the prey species in the collected scats, which varied over time (Appendix B). Considering two types of ungulate prey (wild and domestic) as food categories, a specialized diet in wild prey was observed every year (B standardized < 0.4). On the one hand, when considering the four wild prey species (roe deer, wild boar, red deer, and mountain goat) in the FNB estimation, we observed a broader FNB due to a less strict specialist diet of the wolf in 2019 and 2020. Instead, the wolf’s diet became specialized again, feeding primarily on roe deer and wild boar in 2021, as was the case in 2017 and 2018, when the FNB was narrower. On the other hand, regarding the FNB estimation considering the four domestic preys (cattle, sheep, goat, and horse), a generalist diet was observed in 2017–2018. In 2019, the wolf began to restrict its consumption of a greater variety of domestic livestock prey, and its diet began to be considered as specialist (feeding almost exclusively goat and sheep) from 2020 to 2021.

The FNB estimated according to ingested biomass (kg), obtained from the collected scats, corroborated the results obtained with the calculations based on the frequency of prey occurrence (see Appendix B). A generalist diet was observed in all years except 2021 (year in which FNB was narrower), when two types of prey (wild and domestic) were considered for the calculation of index. The wolf diet was specialist in all years when considering wild ungulates, with the lowest FNB in 2021. FNB estimations from domestic ungulates showed a very specialized diet in cattle in all years, since it was the species that contributed the greatest amount of biomass to the wolf’s diet.

### 3.4. Forest Regions Trends

**Figure 4:**
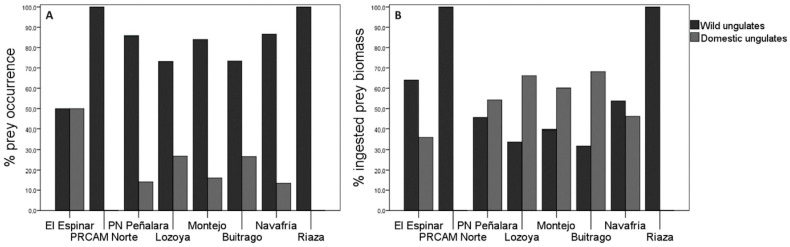
Consumption of wild and domestic ungulates in relation to forest regions: (**A**) % occurrence; (**B**) % ingested biomass. Sample size in El Espinar: *n* = 4; PRCAM Norte: *n* = 2; PN Peñalara: *n* = 262; Lozoya: *n* = 127; Montejo: *n* = 50; Buitrago: *n* = 30; Navafría: *n* = 135; and Riaza: *n* = 3.

The consumption of wild ungulates by wolves was higher compared to domestic ungulates in all forest regions (Figure 4A). Roe deer was the predominant prey in all these regions, except in Navafría, where wild boar was the predominant prey. Among domestic ungulates, cattle were the most consumed animals (see Appendix B). The percentage of occurrence for the different prey species according to the forest regions was statistically significant (χ^2^ = 134.66; df = 56; *p* = 0.025; *n* = 613; see Appendix B). The ingested biomass corresponding to wild ungulates was higher in Navafría than in other forest regions, without considering El Espinar, PRCAM Norte, and Riaza, where the sample size was *n* < 5. (Figure 4B). The greatest biomass contribution to the wolf diet from wild prey species came from wild boar in all forest regions except in Montejo and Buitrago, where the most consumed prey was roe deer (see Appendix B).

The wolves’ diet diversity also varied according to the forest regions. Lozoya accounted for the greatest diversity in diet (H′ = 1.33), unlike PRCAM Norte and Riaza, which were the ones with the lowest diversity (H′ = 0.69 and H′ = 0.00, respectively). All other forest regions showed similar diversity values (El Espinar: H′ = 1.04; Montejo: H′ = 1.17; Navafría: H′ = 1.21; PN Peñarala: H′ = 1.22; and Buitrago: H′ = 1.27). However, the only significant differences were observed between Lozoya and Navafría (t Hutcheson = 2.24; df = 226; *p* = 0.03).

The Ivlev’s index for domestic ungulates according to forest regions showed that the wolves positively selected sheep and goats (El Espinar: D = 0.91; Lozoya: D = 0.76; and Buitrago: D = 0.98) in most regions over cows (El Espinar: D = 0.00; Lozoya: D = 0.04; and Buitrago: D = 0.23). In PN Peñalara, the wolves selected cattle (D = 0.40), but no sheep and goats. In Montejo, the selectivity was similar (cattle: D = 0.52; sheep and goats: D = 0.61). However, in Navafría, the wolves positively selected cattle (D = 0.94) and negatively selected sheep and goats (D = −0.12).

The local authorities gave us information about canid attacks on cattle over 2020 and 2021. The year that ranchers reported the most attacks was 2021. The forest regions where the most attacks were reported were Montejo, PN Peñalara, and Buitrago (Table 2).

## 4. Discussion

The diet of wolves in the Sierra de Guadarrama, Sierra del Rincón, and the surroundings primarily consists of wild ungulates, like other regions in Europe [49,70,77,78]. However, there were differences in wolf diet compared to areas south of the Duero River in the Iberian Peninsula [6]. Our study area is characterized by a multi-prey ecosystem and well-distributed wild ungulates, such as roe deer and wild boar. This could explain why these species were the main prey, while the consumption of domestic livestock, particularly free-roaming cattle, was minimal. Additionally, the larger size of adult cattle makes them a challenging target for wolves, with attacks primarily targeting calves.

### 4.1. General Remarks

Wild boar was the predominant prey in terms of being the most frequently encountered species in the wolf scat samples, followed by roe deer, which is consistent and aligns with Mori et al. [79] in Italy. Wild boar populations in the Iberian Peninsula have increased throughout the region since the start of the 21st century [80,81,82], even at high elevations [83], while roe deer populations are strongly associated with forested areas [84]. This difference in availability distribution explains why the most consumed prey was wild boar, although wolves heavily rely on roe deer as well. Ivlev’s index supports this conclusion, indicating positive selection for roe deer and wild boar compared to other prey species. When considering the percentage of ingested biomass, cattle were the most consumed species, but this finding should be interpreted with caution due to the difference in body size between cattle and other prey species (e.g., cattle outweigh wild boar by a factor of 10). Considering this, the frequency of occurrence should be regarded as a superior metric for assessing carnivore diets, as estimating biomass might lead to overestimating certain larger prey, such as horses or cows.

### 4.2. Seasonal Trends

The consumption of wild ungulates based on the percentage of occurrence of prey was higher in spring and summer (reproductive and breeding season), decreasing its consumption in autumn and winter. In contrast, the consumption of domestic ungulates was higher in autumn and winter. Pups abound in spring and summer and are an easy prey for wolves, given their inexperience [22,85]. The scarcity of this prey species during the colder seasons can compromise cattle, and they may be perceived as more attractive to wolves. Specifically, roe deer was the most consumed prey in all seasons, except spring, when wild boar became the predominant prey, likely due to the high reproductive rate and larger litters (ẋ = 3.5 ind.; [51]) of wild boar compared to roe deer (ẋ = 1.46 ind.; [86]). When considering ingested biomass, cattle consistently contributed the highest percentage in all seasons due to their larger body size. The FNB findings suggest that the wolves exhibited a specialized diet during spring and summer, while showing a more general feeding pattern in autumn and winter. During the reproductive seasons (spring and summer), wolves have a larger pool of prey to choose from, due to the increased population of ungulates resulting from the birthing season. This allows them to selectively target specific prey and specialize their diet according to resource availability. Conversely, in winter, when food availability is limited, the wolf’s diet becomes more generalized, consuming both domestic species and wild ones. This suggests that the wolf in the study area is a facultative specialist species, adapting its feeding behavior depending on the seasons.

### 4.3. Annual Trends

The consumption of wild ungulates was higher than that of domestic ungulates. The wolf’s diet was mainly based on roe deer from 2017 to 2019, while wild boar prevailed in recent years, possibly due to a decrease in roe deer populations (ungulate census of the Nationa Park Sierra de Guadarrama F. Horcajada, unpublished data) since the establishment of wolves in the area. The presence of red deer in the wolf scats in 2019, despite not being generally present in the study area (although it is present in the eastern and southern surroundings), could be attributed to the dispersal behavior of wolves [87,88] or bait placement by hunters [89] or researchers intended for study [44]. The consumption of mountain goats was sporadic, except for a slight increase in 2019. Mountain goats frequent rough areas that are difficult to access and/or guarantee a successful attack from wolves [90]. Wolves prefer steep slopes and open habitats where wild ungulates are more easily detectable and accessible [91], which may explain their lower consumption of mountain goats. The consumption of domestic horse in 2019 was a sporadic event, likely consumed as carrion. Based on FNB, and considering the % occurrence of prey in scats, the wolf followed a specialist diet every year, feeding on wild ungulates instead of domestic ones. However, based on the biomass ingested, the wolf diet could be considered as generalist, except in 2021. This could be due to the different sizes of the preys, as previously discussed.

### 4.4. Forest Regions Trends

The wolves fed mainly on wild ungulates, especially roe deer and wild boar, in all forest regions. Among the species of domestic ungulates, cattle were the most consumed prey. In most of the forest regions, the presence of cattle is greater than that of sheep and goats, except Navafría, where goats and sheep are predominant (INE 2020). Although cattle are more numerous in most forest regions, the prey selection index was higher for sheep and goats. However, in the case of Navafría, where the presence of cattle is lower, the wolves positively selected cattle and avoided sheep and goats.

Finally, an important concern in the study area is the inconsistency between the official data on canid attacks on livestock provided by the Comunidad de Madrid and the findings regarding the wolf’s diet in different forest regions. For instance, the consumption of wild ungulates in PN Peñalara was significantly higher (2020: 78.6%, *n* = 6; 2021: 88.2%, *n* = 45) compared to domestic ungulates (2020: 21.4%, *n* = 3; 2021: 10.2%, *n* = 6 in PN Peñalara; Table 2). In Montejo, where the highest number of attacks was recorded, it paradoxically had one of the lowest consumption rates of domestic ungulates from 2017 to 2019. Despite the lack of diet data for Montejo in 2020–2021, which coincides with the peak number of attacks, the pattern of low domestic ungulate consumption in previous years suggests that the attacks may be primarily caused by other canids such as dogs rather than wolves. The higher number of attacks registered in 2020 and 2021 compared to the detection of domestic ungulate remains in the wolf scats’ similar prey diversity indices in other regions, like Buitrago (H′ = 1.27) and PN Peñalara (H′ = 1.22), further supports this hypothesis. We were unable to draw conclusions about the El Espinar, PRCAM Norte, and Riaza forest regions due to low sample size.

## 5. Conclusions

In conclusion, the diet of the Iberian wolf in the Sierra de Guadarrama National Park, Sierra del Rincón, and neighboring areas is mainly made up of wild ungulate species and a minority of domestic ones. The wolves exhibited selective feeding behavior, preferring roe deer and wild boar while avoiding other ungulates, especially domestic ones. These findings contradict the high number of reported attacks on livestock by forest rangers (Table 2). Roe deer and wild boar were the most frequently preyed upon species in the study area, with their rankings alternating depending on the year. Among domestic ungulates, cattle were the most targeted prey, contributing the highest biomass percentage in the wolf’s diet. The diversity of the wolf’s diet varied seasonally and annually, with a decreasing trend in the consumption of livestock over time. Maintaining a diverse and abundant wild prey population, especially during conflicting seasons when domestic animals are present in the field, can help to reduce or prevent attacks on livestock, as supported by other studies [2,32]. Therefore, we recommend actions that benefit roe deer and wild boar populations, particularly related to forest and hunting management, in addition to a correct knowledge of wild boar population consistence using new technologies such as camera traps, GIS, and remote sensing. These technologies also allow for studying aspects of animals diseases [92]. This study highlights the effectiveness of non-invasive methods for monitoring the wolf’s trophic ecology and obtaining valuable information for species management and conservation. We emphasize the importance of conducting long-term monitoring to collect extensive data, which can provide reliable and precise conclusions. This approach enables the exploration of alternative solutions for conservation conflicts and promotes better coexistence between large predators and humans.

## Figures and Tables

**Table 2:** Number of canid attacks reported by gamekeepers in 2020 and 2021 registered by the Consejería de Medio Ambiente, Vivienda y Agricultura de la Comunidad de Madrid (Counited de Madrid, unpublished data) compared to occurrence of domestic ungulates in scats from 2017 to 2021 and to total scats collected each year in each region.

	Canid Attacks Reported		Domestic Ungulates Occurrence Detected in Scats												Total Scats Collected in Each Region	
	2020	2021	2017		2018		2019		2020		2021		Total			
Forest Region	*n*	*n*	*n*	%	*n*	%	*n*	%	*n*	%	*n*	%	*n*	%	*n*	%
El Espinar	-	-	0	0.0	2	1.3	-	-	-	-	-	-	2	0.3	4	0.7
PRCAM Norte	3	10	-	-	-	-	-	-	0	0.0	0	0.0	0	0.0	2	0.3
PN Peñalara	16	12	1	1.0	7	4.7	20	6.8	3	21.4	6	10.2	37	6.0	262	42.7
Lozoya	8	1	11	11.0	12	8.0	11	3.8	0	0.0	0	0.0	34	5.6	127	20.7
Montejo	25	26	2	2.0	1	0.7	5	1.7	-	-	-	-	8	1.3	50	8.2
Buitrago	13	8	8	8.0	0	0.0	-	-	-	-	-	-	8	1.3	30	4.9
Navafría	-	-	1	1.0	3	2.0	13	4.5	1	7.1	0	0.0	18	2.9	135	22.0
Riaza	-	-	-	-	0	0.0	-	-	-	-	-	-	0	0.0	3	0.5
Total	66	74	23	23.0	25	16.7	49	16.8	4	21.4	6	10.2	107	17.4	613	100
Total scats collected each year			100	16.3	150	24.4	290	47.3	14	2.3	59	9.6	613	100.0	613	100.0

## Data Availability

The data presented in this study are available on request from the corresponding author.

## Appendix A

### Appendix A.1. Mapping Using Kernel Densities

Kernel density is based on two defining parameters: the radius of influence (*R*) and the estimation function (*k*). The radius of influence was defined as the area centered on the estimation point *P* that indicates how many events contribute to the estimate of the intensity function λ. The estimation function (*k*) takes care of the properties to smooth the density calculated by the Kernel technique and was calculated through the formula:

$\lambda \left(P\right)\sum_{i=1}^{n}\;\frac{1}{R^{2}}k\frac{\left(P-Pi\right)}{R}\;$ where *k* is a bivariate and symmetric Kernel function called the estimation or smoothing function and the parameter *R* > 0 is known as the width of the band (or radius of influence) and determines the degree of smoothing. This is the radius of a disk centered at *P* (*P* represents any location in *R*), where *P_i_* will contribute significantly [65,66,93].

### Appendix A.2. Equation of Floyd et al. (1978) [66], Revised and Adjusted by Weaver (1993) [67]

This equation (y=0.439+0.008x) describes the relationship between the body mass of the prey (kg) and the mass of the prey consumed (kg) per scat collected, where y is the mass of prey consumed per scat collected and x is the body mass average of an individual of a given prey species. Average mass was used because the age of the prey consumed leads to an overestimation of the contribution of smaller prey species to the diet due to their greater amount of hair and other indigestible matter per unit of body mass, which produces more scats per unit mass of prey consumed [67]. The estimate of the total biomass of each of the prey species in the scats was obtained by multiplying the calculated value of y by the number of collected scats containing each prey species. The biomass percentage of each prey item consumed was calculated by dividing the total weight of a particular prey item consumed by the weight of all the mammalian prey items consumed.

### Appendix A.3. Shannon Diversity Index Calculation

The Shannon diversity index was calculated to estimate dietary diversity according to seasonality and years.

$$\mathrm{Shannon}\;\mathrm{diversity}\;\mathrm{index}:\;H^{\prime }=-\sum_{i=1}^{s}p_{i}{log}_{2}p_{i}$$

Significant differences between pairs of Shannon indices were tested using Hutcheson’s *t*-statistic. Hutcheson’s *t*-test is a modified version of the classical *t*-test that provides a way of comparing two samples using the variance of the Shannon index (Hutcheson 1970) [70].

$$\mathrm{Hutcheson}’s\;t-\mathrm{test}:\;t=\frac{H_{a}H_{b}}{\sqrt{S_{H_{a}}^{2}-S_{H_{b}}^{2}}}$$

In the formula, *H* represents the Shannon diversity index (Shannon 1948) for each of the two samples (subscript *a* and *b*). The lower part of the formula refers to the variance of each of the samples. The calculation of the variance of the Shannon diversity is performed using the formula shown below:

$$S_{H}^{2}=\frac{\sum p{\left(\ln p\right)}^{2}-{\left(\sum p\ln p\right)}^{2}}{N}+\frac{s-1}{2N^{2}}$$

In the formula, *S* is the total number of species, while *N* is the total abundance. *p* is the proportion that each species makes up with respect to the total.

### Appendix A.4. Niche Breadth (Levin’s Index) Calculation

The niche breadth and niche overlap of the wolf’s diet were calculated according to the frequency of occurrence for the prey consumed and the biomass ingested over the seasons and years. Levin’s food niche breadth (FNB) Index (Levins 1968) [71] was used to quantitatively measure specialization in the composition of the wolf’s diet.

$$\mathrm{Levins}’s\;\mathrm{index}:\;B=\frac{1}{\sum p_{i}^{2}}$$

In the formula, B represents the food niche breadth of the wolf and $p_{i}$ the proportion of contribution of each group of wolf prey in the total biomass of food consumed by the canid (Nowak et al., 2011). The Levins’s index was standardized with the following equation to express the level of specialization on a scale from 0 to 1.

$$\mathrm{Levin}’s\;\mathrm{index}\;\mathrm{standardized}:\;B_{A}=\frac{B-1}{n-1}$$

where $B_{A}=0$ represents a high specialization and $B_{A}=1$ represents the equitable consumption of all prey. In the formula, $B_{A}$ represents the Levin’s index standardized, B represents the Levin’s index, and n represents the number of prey species consumed by the wolf (Müller et al., 2006). If the values obtained are <0.6, this means that the diet analyzed presents a low diversity of prey, considering the species under study a specialist predator. On the other hand, if the values are >0.6, they indicate that the species shows generalist eating habits, that is, it is a generalist predator (Krebs 1999; Cruz-Escalona et al., 2000).

We calculated the FNB in each of the four seasons and in each of the five years using both the data in the form of % occurrence and % biomass ingested. This index was calculated in three ways:

1. Wild/Dom: two categories of *p_i_*, one corresponding to the total proportion of wild ungulates and the other corresponding to the total proportion of domestic ungulates that appeared in the wolf feces in each season.
2. Wild/Wild: four categories of *p_i_*, corresponding to the proportions of each wild ungulate species (roe deer, red deer, wild boar, and mountain goat) appearing in the wolf feces in each season.
3. Dom/Dom: four categories of *p_i_*, corresponding to the proportions of each species of domestic ungulate (cattle, sheep, goat, and horse) that appeared in the wolf feces in each season.

### Appendix A.5. Ivelev’s Electivity Index Calculation

Ivlev’s electivity index modified by Jacobs (1974) [72] was calculated to assess whether the wolves selected prey positively or negatively. This index was applied, on the one hand, to evaluate the selection of prey throughout the study area and, on the other hand, to evaluate the selection among domestic ungulates in terms of forest region.

$$\mathrm{Ivlev}’s\;\mathrm{index}:\;D=\frac{\left(r_{i}-p_{i}\right)}{\left(r_{i}+p_{i}-2r_{i}\cdot p_{i}\right)}$$

In the formula, $r_{i}$ is the proportion of a given prey species and $p_{i}$ is the proportion of available prey *i* in the study area. This index generates values ranging from −1 to 1. Negative values indicate prey inaccessibility or the total avoidance of a species, a value of 0 indicates random prey consumption or no selection, and positive values indicate the selection of a specific prey item.

## Appendix B

**Table A1:** Seasonal Variation of Wolf Diet (*n* = 637; pooled years).

			Prey Occurrence		Ingested Biomass	
Prey		Season	*n*	%	Kg	%
Wild ungulates	Roe deer	Autumn	83	43.9	141.4	13.2
		Winter	49	44.1	83.5	20.1
		Spring	76	38.0	129.4	10.7
		Summer	73	53.3	124.3	15.5
	Red deer	Autumn	1	0.5	5.0	0.5
		Winter	0	0.0	5.0	1.2
		Spring	1	0.5	5.1	0.4
		Summer	0	0.0	0.0	0.0
	Wild boar	Autumn	54	28.5	233.2	21.8
		Winter	38	34.2	164.1	39.5
		Spring	86	43.0	371.4	30.8
		Summer	38	27.7	164.1	20.5
	Mountain goat	Autumn	7	3.7	23.2	2.2
		Winter	1	0.9	3.3	0.8
		Spring	4	2.0	13.3	1.1
		Summer	7	5.1	23.2	2.9
	Total	Autumn	145	76.7	402.8	37.7
		Winter	88	79.3	255.9	61.6
		Spring	167	83.5	519.2	43.1
		Summer	118	86.1	311.6	38.9
Domestic ungulates	Cattle	Autumn	18	9.5	630.7	59.1
		Winter	4	3.6	140.2	33.7
		Spring	19	9.5	665.7	55.2
		Summer	13	9.5	455.5	56.9
	Domestic goat	Autumn	12	6.3	20.34	1.9
		Winter	3	2.7	5.1	1.2
		Spring	8	4.0	13.6	1.1
		Summer	5	3.6	8.5	1.1
	Sheep	Autumn	8	4.2	14.3	1.3
		Winter	3	2.7	14.2	3.5
		Spring	4	2.0	7.1	0.6
		Summer	0	0.0	0.0	0.0
	Horse	Autumn	0	0.0	0.0	0.0
		Winter	0	0.0	0.0	0.0
		Spring	0	0.0	0.0	0.0
		Summer	1	0.7	24.8	3.1
	Unidentified livestock	Autumn	6	3.2	0.0	0.0
		Winter	13	11.7	0.0	0.0
		Spring	2	1.0	0.0	0.0
		Summer	0	0.0	0.0	0.0
	Total	Autumn	44	23.3	665.3	62.3
		Winter	23	20.7	159.5	38.4
		Spring	33	16.5	686.4	56.9
		Summer	19	13.9	488.8	61.1

**Table A2:** The percentage of occurrence of wild and domestic ungulates according to seasons. The statistical results according to season comparing the consumption of domestic or wild ungulates, respectively.

Season	Fecal Samples Collected (*n*)	% Wild Ungulates Occurrence	% Domestic Ungulates Occurrence	Statistical Results in Each Season (Wild vs. Domestic)
Autumn	189	76.7	23.3	χ^2^ = 53.97; df = 1; *p* = 0.001
Winter	111	79.3	20.7	χ^2^ = 8.06; df = 1; *p* = 0.001
Spring	200	83.5	16.5	χ^2^ = 89.78; df = 1; *p* = 0.001
Summer	137	86.1	13.9	χ^2^ = 71.54; df = 1; *p* = 0.001
**Statistical results in all seasons** **(wild species vs. domestic species)**		χ^2^ = 16.81; df = 9; *p* = 0.037, *n* = 518	χ^2^ = 43.59; df = 12; *p* = 0.001, *n* = 119	

**Table A3:** The food niche breadth (A) of the wolf’s diet according to seasons.//Number of preys = number of prey species found in collected wolf scats.//Wild/Dom calculates the indicators based on two types of prey species (wild species and domestic species); Wild/Wild calculates the indicators based on four species of wild prey (roe deer, wild boar, red deer, and mountain goat); and Dom/Dom calculates the indicators based on four domestic prey species (cattle, sheep, domestic goat, and horse).

	Occurrence (n)			Biomass (Kg)		
	*B′*			*B′*		
	Wild/Dom	Wild/Wild	Dom/Dom	Wild/Dom	Wild/Wild	Dom/Dom
Autumn	0.31	0.37	0.57	0.90	0.40	0.00
Winter	0.21	0.33	0.64	0.90	0.30	0.10
Spring	0.26	0.37	0.44	1.00	0.20	0.00
Summer	0.24	0.35	0.28	0.90	0.40	0.00
Number of preys	2	4	4	2	4	4

**Table A4:** Yearly changes in Iberian wolf diet (*n* = 637; pooled seasons).

			Prey Occurrence		Ingested Biomass	
Prey		Years	*n*	%	Kg	%
Wild ungulates	Roe deer	2017	53	48.7	90.3	20.0
		2018	87	51.5	148.2	20.4
		2019	119	41.5	202.7	10.8
		2020	9	30.0	15.3	5.3
		2021	13	31.0	22.1	16.5
	Red deer	2017	0	0.0	0.0	0.0
		2018	0	0.0	0.0	0.0
		2019	2	0.7	10.1	0.6
		2020	0	0.0	0.0	0.0
		2021	0	0.0	0.0	0.0
	Wild boar	2017	31	28.4	133.9	29.6
		2018	47	27.8	203.0	27.9
		2019	100	34.8	431.9	23.0
		2020	14	46.7	60.5	20.9
		2021	24	57.1	103.7	77.1
	Mountain goat	2017	0	0.0	0.0	0.0
		2018	1	0.6	3.3	0.5
		2019	17	5.9	56.4	3.0
		2020	1	3.3	3.3	1.1
		2021	0	0.0	0.0	0.0
	Total	2017	84	77.1	224.2	49.6
		2018	135	79.9	354.5	48.8
		2019	238	82.9	701.1	37.4
		2020	24	80.0	79.1	27.3
		2021	37	88.1	125.8	93.6
Domestic ungulates	Cattle	2017	6	5.5	210.2	46.5
		2018	10	5.9	350.4	48.3
		2019	32	11.2	1121.3	59.8
		2020	6	20.0	210.2	72.7
		2021	0	0.0	0.0	0.0
	Domestic goat	2017	6	5.5	10.2	2.3
		2018	8	4.7	13.5	1.9
		2019	11	3.8	18.7	0.9
		2020	0	0.0	0.0	0.0
		2021	3	7.1	5.1	3.8
	Sheep	2017	4	3.7	7.1	1.6
		2018	4	2.4	7.1	0.9
		2019	5	1.7	8.9	0.5
		2020	0	0.0	0.0	0.0
		2021	2	4.8	3.6	2.7
	Horse	2017	0	0.0	0.0	0.0
		2018	0	0.0	0.0	0.0
		2019	1	0.3	24.8	1.6
		2020	0	0.0	0.0	0.0
		2021	0	0.0	0.0	0.0
	Unidentified livestock	2017	9	8.3	0.0	0.0
		2018	12	7.1	0.0	0.0
		2019	0	0.0	0.0	0.0
		2020	0	0.0	0.0	0.0
		2021	0	0.0	0.0	0.0
	Total	2017	25	22.9	227.5	50.4
		2018	34	20.1	371.0	51.1
		2019	49	17.1	1173.7	62.6
		2020	6	20.0	210.2	72.7
		2021	5	11.9	8.7	6.4

**Table A5:** The percentage of occurrence of wild and domestic ungulates according to years. The statistical results according to year comparing the consumption of domestic and wild ungulates separately.

Year	Fecal Samples Collected (*n*)	% Wild Ungulates Occurrence	% Domestic Ungulates Occurrence	Statistical Results in Each Year (Wild vs. Domestic)
2017	109	77.1	22.9	χ^2^ = 31.94; df = 1; *p* = 0.001
2018	169	79.9	20.1	χ^2^ = 60.36; df = 1; *p* = 0.001
2019	287	82.9	17.1	χ^2^ = 124.46; df = 1; *p* = 0.001
2020	30	80.0	20.0	χ^2^ = 10.80; df = 1; *p* = 0.001
2021	42	88.1	11.9	χ^2^ = 24.38; df = 1; *p* = 0.001
**Statistical results in all years** **(wild species vs. domestic species)**		χ^2^ = 16.81; df = 9; *p* = 0.037, n = 518	χ^2^ = 43.59; df = 12; *p* = 0.001, n = 119	

**Table A6:** The food niche breadth (A) of wolf diet according to years.//Number of preys = number of prey species found in collected wolf scats.//Wild/Dom calculates the indicators based on two types of prey species (wild species and domestic species); Wild/Wild calculates the indicators based on four species of wild prey (roe deer, wild boar, red deer, and mountain goat); Dom/Dom calculates the indicators based on four domestic prey species (cattle, sheep, domestic goat, and horse).

	Occurrence (n)			Biomass (Kg)		
	*B′*			*B′*		
	Wild/Dom	Wild/Wild	Dom/Dom	Wild/Dom	Wild/Wild	Dom/Dom
2017	0.22	0.29	0.63	1.00	0.30	0.10
2018	0.21	0.28	0.56	1.00	0.30	0.00
2019	0.32	0.44	0.35	0.90	0.40	0.00
2020	0.33	0.46	0.00	0.70	0.20	0.00
2021	0.18	0.27	0.30	0.10	0.10	0.30
Number of preys	2	4	4	2	4	4

**Table A7:** A comparison of the compositions of the Iberian wolf diet between forest regions based on 637 scats. The ingested prey biomass (in kg) was calculated using body masses.

			Prey Occurrence		Ingested Biomass	
Prey		Forest Region	*n*	%	Kg	%
Wild ungulates	Roe deer	El Espinar	1	25.0	1.7	18.1
		PRCAM Norte	1	50.0	1.7	28.3
		PN Peñalara	114	43.5	194.1	13.2
		Lozoya	62	48.9	105.6	15.1
		Montejo	30	60.0	51.1	21.4
		Buitrago	16	53.3	27.2	16.3
		Navafría	46	34.1	78.3	11.0
		Riaza	3	100.0	5.1	100
	Red deer	El Espinar	0	0.0	0.0	0.0
		PRCAM Norte	0	0.0	0.0	0.0
		PN Peñalara	1	0.4	5.0	0.4
		Lozoya	0	0.0	0.0	0.0
		Montejo	0	0.0	0.0	0.0
		Buitrago	0	0.0	0.0	0.0
		Navafría	1	0.7	5.0	0.7
		Riaza	0	0.0	0.0	0.0
	Wild boar	El Espinar	1	25.0	4.3	45.9
		PRCAM Norte	1	50.0	4.3	71.7
		PN Peñalara	106	40.5	457.8	31.2
		Lozoya	28	22.0	120.9	17.3
		Montejo	4	8.0	17.3	7.3
		Buitrago	6	20.0	25.9	15.5
		Navafría	67	49.6	289.4	40.7
		Riaza	0	0.0	0.0	0.0
	Mountain goat	El Espinar	0	0.0	0.0	0.0
		PRCAM Norte	0	0.0	0.0	0.0
		PN Peñalara	4	1.5	13.3	0.9
		Lozoya	3	2.4	10.0	1.4
		Montejo	8	16.0	26.6	11.1
		Buitrago	0	0.0	0.0	0.0
		Navafría	3	2.2	9.9	1.4
		Riaza	0	0.0	0.0	0.0
	Total	El Espinar	2	50.0	6.0	64.0
		PRCAM Norte	2	100.0	6.0	100.0
		PN Peñalara	225	85.9	670.2	45.7
		Lozoya	93	73.2	236.5	33.8
		Montejo	42	84.0	95.0	39.8
		Buitrago	22	73.4	53.1	31.8
		Navafría	117	86.6	382.6	53.8
		Riaza	3	100.0	5.1	100.0
Domestic ungulates	Cattle	El Espinar	0	0.0	0.0	0.0
		PRCAM Norte	0	0.0	0.0	0.0
		PN Peñalara	22	8.4	770.9	52.5
		Lozoya	12	9.4	420.5	60.0
		Montejo	4	8.0	140.2	58.8
		Buitrago	3	10.0	105.1	63.0
		Navafría	9	6.7	315.3	44.3
		Riaza	0	0.0	0.0	0.0
	Domestic goat	El Espinar	2	50.0	3.4	36.0
		PRCAM Norte	0	0.0	0.0	0.0
		PN Peñalara	9	3.4	15.3	1.0
		Lozoya	6	4.7	10.2	1.4
		Montejo	1	2.0	1.7	0.7
		Buitrago	4	13.3	6.8	4.1
		Navafría	7	5.3	11.9	1.7
		Riaza	0	0.0	0.0	0.0
	Sheep	El Espinar	0	0.0	0.0	0.0
		PRCAM Norte	0	0.0	0.0	0.0
		PN Peñalara	6	2.3	10.7	0.7
		Lozoya	5	3.9	8.9	1.3
		Montejo	1	2.0	1.8	0.7
		Buitrago	1	3.3	1.9	1.1
		Navafría	1	0.7	1.8	0.2
		Riaza	0	0.0	0.0	0.0
	Horse	El Espinar	0	0.0	0.0	0.0
		PRCAM Norte	0	0.0	0.0	0.0
		PN Peñalara	0	0.0	0.0	0.0
		Lozoya	1	0.9	24.8	3.5
		Montejo	0	0.0	0.0	0.0
		Buitrago	0	0.0	0.0	0.0
		Navafría	0	0.0	0.0	0.0
		Riaza	0	0.0	0.0	0.0
	Unidentified livestock	El Espinar	0	0.0	-	-
		PRCAM Norte	0	0.0	-	-
		PN Peñalara	0	0.0	-	-
		Lozoya	10	7.9	-	-
		Montejo	2	4.0	-	-
		Buitrago	0	0.0	-	-
		Navafría	1	0.7	-	-
		Riaza	0	0.0	-	-
	Total	El Espinar	2	50.0	3.4	36.0
		PRCAM Norte	0	0.0	0	0.0
		PN Peñalara	37	14.1	796.9	54.3
		Lozoya	34	26.8	464.4	66.2
		Montejo	8	16.0	143.7	60.2
		Buitrago	8	26.6	113.8	68.2
		Navafría	18	13.4	329.0	46.2
		Riaza	0	0.0	0.0	0.0

**Table A8:** The percentage of occurrence of wild and domestic ungulates according to forest regions. The statistical results according to forest region comparing the consumption of domestic and wild ungulates.

Forest Region	Fecal Samples Collected (*n*)	% Wild Ungulates Occurrence	% Domestic Ungulates Occurrence	Statistical Results (Wild vs. Domestic)
El Espinar	4	50.0	50.0	-
PRCAM Norte	2	100.0	0.0	-
PN Peñalara	262	85.9	14.1	χ^2^ = 134.90; df = 1; *p* = 0.001
Lozoya	127	73.2	26.8	χ^2^ = 27.40; df = 1; *p* = 0.001
Montejo	50	84.0	16.0	χ^2^ = 23.12; df = 1; *p* = 0.001
Buitrago	30	73.4	26.6	χ^2^ = 6.53; df = 1; *p* = 0.11
Navafría	135	86.6	13.4	χ^2^ = 72.6; df = 1; *p* = 0.001
Riaza	3	100.0	0.0	-
**Statistical results** **(wild species vs. domestic species)**		χ^2^ = 69.68; df = 21; *p* = 0.038, *n* = 506	χ^2^ = 30.94; df = 20; *p* = 0.065, *n* = 107	
